# Animal Models in Osteosarcoma

**DOI:** 10.3389/fonc.2014.00189

**Published:** 2014-07-18

**Authors:** Maria V. Guijarro, Steven C. Ghivizzani, C. Parker Gibbs

**Affiliations:** ^1^Department of Orthopaedics and Rehabilitation, University of Florida, Gainesville, FL, USA

**Keywords:** osteosarcoma, conditional mouse models, germ-line mouse models, animal models, p53, RB

## Abstract

Osteosarcoma (OS) is the most common non-hematologic primary tumor of bone in children and adults. High-dose cytotoxic chemotherapy and surgical resection have improved prognosis, with long-term survival for non-metastatic disease approaching 70%. However, most OS tumors are high grade and tend to rapidly develop pulmonary metastases. Despite clinical advances, patients with metastatic disease or relapse have a poor prognosis. Toward a better understanding of the molecular pathogenesis of human OS, several genetically modified OS mouse models have been developed and will be reviewed here. However, better animal models that more accurately recapitulate the natural progression of the disease are needed for the development of improved prognostic and diagnostic markers as well as targeted therapies for both primary and metastatic OS.

## Introduction

Osteosarcoma (OS) is a highly malignant form of bone cancer characterized by osteoid production. Although OS comprises <1% of cancers diagnosed in the United States, it is the most common primary malignancy of the bone (1, 2). It occurs predominantly after the first decade of life during periods of skeletal growth, with a second peak incidence in the geriatric patient population (1, 3). The vast majority of OS in children, adolescents, and young adults is high grade and begins in the intramedullary space of metaphyseal locations in long bones of the lower extremity. This suggests a relationship with active growth plates. After a low incidence in individuals between 25 and 59 years of age, the incidence of OS rises again in individuals over 60 years of age, and is most often associated with Paget’s disease or radiation exposure (1, 2). This may suggest that the underlying pathogenesis is not identical in young and older patients. Conventional OS presents in three major subtypes based on histological classification: osteoblastic, fibroblastic, and chondroblastic. Osteoblastic is the most common (around 60%) with fibroblastic and chondroblastic being equally represented (4).

Osteosarcoma is characterized by a local invasion of bone and soft tissue, loss of the function of the affected extremity, and distant metastasis, most often to the lung (90%). Metastases are also found in bone (8–10%) and rarely in lymph nodes (5). Treatment involves aggressive removal of the primary tumor to afford local control via limb sparing surgery or amputation. Systemic chemotherapy (both prior to and after tumor removal) is used to suppress development of metastasis and effect cure. The most common chemotherapy regimens comprise the drugs, cisplatin, doxorubicin, and high-dose methotrexate in combination (6–8). Although chemotherapy slows tumor growth, it can induce cardiomyopathy, hearing loss, and risk of secondary malignancy (8, 9). In patients without metastases at the time of diagnosis (80–90%), surgical treatment in combination with chemotherapy has resulted in long-term survival rates that approach 70%. In contrast, for patients with established metastases there is currently no reliable therapeutic option to provide long-term tumor control. Despite intensive efforts to improve both chemotherapeutics and surgical management, 40% of all OS patients succumb to the disease. Specifically, the clinical outcome for metastatic OS remains poor; fewer than 30% of patients who present metastases survive 5 years after initial diagnosis. Therefore, there is an urgent need for the development of novel therapeutics for OS agents with increased capacity to eliminate systemic tumor burden as well as reduced toxicity in healthy tissues.

## Etiology of OS

Osteosarcoma is characterized by a complex karyotype and a lack of recurrent translocations. Genetic approaches have identified several genes of potential importance in the development and progression of the disease (10–12). However, the widespread chromosomal alterations of the OS genome have limited the interpretation of these findings. Genetic alterations of OS are usually sporadic though genetic predisposition has been documented in patients with Li-Fraumeni and retinoblastoma syndrome. Somatic deletions and point mutations in *P53* occur in approximately 50% of human OS (13–16) and half of those mutations are associated with loss of the remaining allele (14). Additionally, almost 70% of OS have at least one *RB* allele alteration (17, 18). Homozygous deletions of *RB* are seen in 23% of tumors, while point mutations appear in 6% (18, 19). In addition, numerous alterations that disrupt the *RB* pathway have also been reported; for example, the loss of function at the *INK4a/ARF* locus and the amplification of *CDK4* have been found to occur (one or the other) in 22% of OS (20–22). The prevalence of these alterations would suggest that the deregulation of both G1/S and G2/M checkpoint in the cell cycle are a common event in OS.

For this, a tumor of unknown origin, chaotic genetics, early onset, and aggressive behavior, there is a need for more representative models to learn more about the biology of OS.

## Animal Models in OS

Animal models hold significant promise in increasing our understanding of the genetic basis of OS and more importantly, in advancing preclinical studies aimed to the rational development of new therapeutic approaches as well as their validation prior to clinical trials.

In order for any animal model of human disease to be useful and informative, it is preferable to accurately recapitulate the natural course of the disease. Unfortunately, the etiology and pathogenesis of OS are not completely understood; therefore, the establishment and induction of representative experimental models are challenging and incomplete. Currently, there is not a robust animal model of OS that fully represents its biological and clinical features. The ideal would be one in which there was a naturally occurring primary bone lesion and spontaneous pulmonary metastases. To date, the major species used to generate OS models are mouse and rat; however, OS arising in dogs is also of note as a validated model of spontaneous OS.

Many aspects of the biology of the disease have been determined from a variety of animal model approaches. Genetically modified mouse models of OS have given the field much insight. However, spontaneous OS, secondary OS as a consequence of animals receiving radiation, human and murine OS cell lines, and xenotransplantation studies are also important to understand the biology of this malignancy.

## Canine Models

Spontaneous OS is much more common in large dogs than in humans, making the dog an attractive candidate model to study human disease (23). Canine OS is indistinguishable from human tumors at the histological and gene expression levels (24–27). The primary differences between the two are the age of development and the prevalence of the disease. In dogs, OS is a disease of older, large breed dogs (6–12 years of age), and it is estimated that over 10,000 cases occur annually in the United States. The median disease-free interval following surgery alone is 4 months, and after surgery with chemotherapy, 13 months. This high prevalence and the relatively rapid rate of disease progression provide the opportunity to model metastasis development and progression and evaluate novel treatment options in a relatively short period of time (28–32). Many of the genes involved in human OS pathogenesis appear to participate in canine OS, including *P53, RB*, and *PTEN* (33–36).

Although canine OS serves as an excellent comparative tumor model for human OS, there are some limitations to be considered. First, OS affects skeletally mature, geriatric dogs, which is different from humans where the peak of incidence occurs during adolescence. Second, some breeds have specific heritable germ-line mutations in certain genes that may influence OS biology, progression, and response to treatment without driving the initiation of the disease (37).

## Secondary OS after Radiation

The development of rodent OS models began with the exposure of rats and mice to chemical and radioactive carcinogens (38–40). Of note, among those was the development of OS in rats treated with P^32^-orthophosphate, which resulted in a high incidence (41). These models yielded tumors that histologically resembled the human cancer and produced cell lines that complement human OS studies (42). Despite the high penetrance of the models, their relevance remains unclear since the majority of OS in humans is sporadic, while the carcinogen-induced murine model is more representative of a therapy induced disease.

## Xenotransplantation Studies

There is a significant amount of literature related to the development and use of xenograft and allograft models of human and murine OS cells injected into immunocompromised mice. The injected cells form a solid tumor locally grown within days or weeks after implantation (42, 43). The use of these systems has become a prominent tool in current oncological research due to the quick onset, its affordable cost, and ease of handling and maintenance. In addition, OS donor-derived cells may metastasize to the lungs, providing an opportunity to investigate primary and secondary tumor growth. The principal limitation is that the approach uses fully developed OS cells and therefore does not provide information about the initiation of the tumor and its etiology. Furthermore, since the tumor microenvironment can contribute significantly to the tumor behavior, such interactions may be lost when establishing the disease by direct introduction into a recipient animal (44–46). In certain circumstances, the injected cell line may not be metastatic in the rodent context, making it impossible to study the dissemination of the disease. Despite these limitations, many groups have successfully used this model to identify factors involved in OS migration (47, 48) and more importantly for screening drugs with tumoricidal potential (49). Distinct advantages of the subcutaneous cell suspension injection model are high rate of incidence and reproducibility that allows for accurate titration of cell numbers in the inoculum to quantify tumorigenic potential of the injected cells.

A variation of injecting cell suspensions into recipient animals is to *transplant* pieces of tumor directly harvested from the patient. The advantage is that the human malignant cells can grow in its native environment maintaining the heterogeneity that may be required for their proliferation, which in some reports has been shown to enhance tumor growth and metastasis. With the use of cell suspension and transplants, murine host cells can infiltrate the tumor, possibly influencing the activities of the tumor cells, and in some cases, cells of the rodent host can overgrow the human cell population (50). Alternatively orthotopic, *intratibial implantation* of OS cells has been shown to induce OS at local and metastatic sites (proximal tibia and lung) (43, 51–53). This approach allows the study of primary tumor formation within a more native context as well as the early stages of metastatic progression of OS, thereby reconstituting the entire metastatic process. Its use, however, is limited by a lack of reproducibility due in part to the technical skill required to perform the implantation and the associated lack of quantifiable inoculum.

## Genetically Engineered Mouse Models

Of the sarcomas with complex karyotypes, OS is one of the most well-studied as exemplified by the development of numerous mouse models available for this disease. The ability to alter specifically the expression of individual genes (by loss or gain of function) became available in the mouse with the evolution of gene targeting technologies (54, 55).

Many murine OS models have been developed to recapitulate the *P53* and *RB* mutations in hereditary and sporadic human OS. *Germ-line* deletion of *P53* resulted in an OS incidence of 4% in homozygous *P53* null mice (56) and 25% in heterozygous *P53* mice (57), underlying the importance of altered *P53* in driving OS. This unexpected ratio of tumor formation, though, is likely due to the early lethality seen in the homozygous null population. Further, the rapid development, the higher incidence of other tumors (mostly lymphomas), and the long latency of OS (58) necessitate the sacrifice of the mice before OS onset, hampering in many cases the utility of these models. The role of *P53* was further highlighted by tumor analysis of *P53* knock-in mice containing a mutant copy of *P53R172H* (corresponding to the *R175H* hot-spot mutation in humans) that not only develop primary tumors but also metastasize to the lungs as well as other organs (59, 60). Conversely, mice with germ-line deletions of *Rb* did not develop OS: homologous deletion of *Rb* is embryonic lethal and the heterozygotes are not predisposed to OS (61, 62).

**Figure 1:**
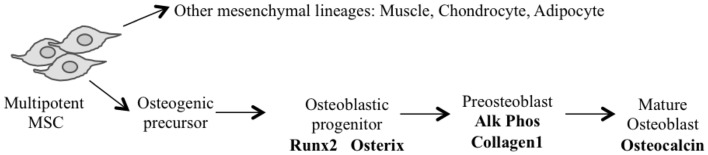
**Model of osteoblast differentiation and putative stage of *Cre* expression is shown**.

The application of conditional gene regulation and the availability of tissue specific *Cre expressing mouse lines* (63) have greatly enhanced our ability to generate specific models of mesenchymal osteogenic lineage that more faithfully resemble human OS (55, 64). The majority of these models have used the loss of *P53* with or without the disruption of the *Rb* pathway to generate penetrant OS models (54). They use conditional gene deletion approaches restricted to multipotent mesenchymal progenitors, early committed osteoblasts (pre-osteoblasts) and the osteoblast population (Figure 1) (Table 1).

**Table 1:** **Summary of genetically modified OS murine models**.

Cell	Cre	Gene	OS penetrance (%)	Other tumors	Metastatic disease
MSC/skeletal	*Prx-1*	p53^fl/+^	22 (65)		
Progenitors		p53^fl/fl^	61 (65); 62(66)	PDS (32%), LY (3%), LPS (3%); RMS (15%), PDS (12%)	Yes (24%)
		p53^fl/fl^-Rb^fl/+^	92 (66)	RMS (9%), PDS (18%), HIB (4%)	
		p53^fl/fl^-Rb^fl/fl^	18 (65); 29 (66)	PDS (57%), LY (14%); RMS (12%), PDS (3%), HIB (91%)	
Pre-osteoblasts	*Osx*	p53^fl/fl^	100 (67); 100 (68)		Yes (32%); yes (40%)
		p53^fl/fl^-Rb^fl/+^	53 (67); 100 (68)		
		p53^fl/fl^-Rb^fl/fl^	72 (67); 100 (68)	Multiple tumors per animal; concurrent HIB (20–25%)	Yes (37%)
		shp53	100 (69)	0%	Yes (83.33%)
		shp53-Rb^fl/+^	100 (69)	0%	Yes (58.82%)
		shp53-Rb^fl/fl^	100 (69)	0%	Yes (85.72%)
	*Col1α1–3.6*	p53^fl/fl^	60 (70)		
Osteoblasts	*Col1α1–2.3*	p53^fl/fl^	85 (65)		
	*Og2*	SV40 Tag	100 (71)		Yes (90%)

*LPS, liposarcoma; LY, lymphoma; RMS, rhabdomyosarcoma; PDS, poorly differentiated sarcoma; HIB, hibernomas*.

Using *Cre* recombinase activated by the gene promoter of Paired related homebox 1 (*Prx1-Cre)* (72) that deletes *LoxP* flanked alleles in the early limb mesenchyme (multipotential cells), 22% of mice with *P53*-mediated heterozygosity developed OS. Not surprisingly, homozygous deletion of *P53* had a threefold increase in OS incidence over the heterozygous animals. In contrast, the deletion of *Rb* in the mesenchymal *Prx* expressing progenitors did not produce any OS tumors (65, 66). Interestingly, the highest incidence (92%) of OS occurred with the combined deletion of one allele of *Rb* with homozygous *P53* deletion (66). Homozygous deletion of both genes resulted in more non-specific tumor formation with only 18% OS tumors and the remainder being poorly differentiated soft tissue sarcomas (PD-STS) and lymphoma (65, 66).

For a more restricted deletion of genes in the *osteoblast lineage*, promoters of genes ranging from those expressed early in the commitment of progenitors as Osterix 1 and Collagen1 α1–3.6 to those expressed in more lineage-restricted osteoblast precursors such as Collagen1 α1–2.3 and osteocalcin (*Og2*) have been used. Development of OS with a penetrance of 100% (67, 68) has been observed following osteoblast specific deletion of *P53* using Osterix-mediated *Cre* expression (*Osx-Cre*) (73). As with mesenchymal progenitors, *Rb* deletions have no effect and combined deletion of *Rb* and *P53* in osteoblasts once again generated fibroblastic or undifferentiated OS with high penetrance (100%) (67, 68). Potential translational utility is the existence of short-latency spontaneous metastatic OS similar to human tumors in which cells are arrested in their differentiation (67, 68). Although the greatest proportion of tumors was OS when *P53* was conditionally deleted, neuroendocrine tumors and hibernomas were also reported to be generated in several mice (67, 68). However, Walkley et al. enriched the C57BL/6 background of the mouse strain and the percentage of hibernomas was reduced, suggesting a possible impact of mouse strains in the phenotype observed (69). A recent study in mice that expressed SV40 T/t antigen (Tag) in mature osteoblasts under the *Og2* (74) showed OS with complete penetrance (71) and 90% incidence of lung metastases. Further analysis of the tumors derived from this model revealed a recurrent genomic deletion of the *Prkar1a* gene in a specific subset also in human OS. Transgenic *sh*RNA has been used to specifically knock down *P53* (rather than delete) using the *Osx-Cre* transgene (69). These mice develop osteoblastic OS with a 100% penetrance, and although they have a longer latency to tumor onset, they more often develop in long bones and are highly metastatic (lung and liver), features similar to human OS. This model has not developed any non-OS tumors.

Independent of the stage of development in which *Cre* becomes active, the latency of OS is essentially the same when comparing either *P53* alone or in combination with *Rb*. The use of *Cre* in more primitive cells (*Prx*), however, leads to the development of tumors of other mesenchymal lineages at higher frequency.

Possibly providing insight into the initiating events of OS (70), a prominent cellular feature of conditional inactivation of *P53* in osteoblastic progenitors is the hyperproliferation of osteoblasts prior to tumor formation. *Rb* has been proposed to have a role in influencing late osteoblast differentiation by interacting with *Runx2* (75). However, a number of independent studies have shown that the removal of *Rb* alone is not sufficient to induce OS. The different experimental approaches strongly suggest that mutation in the *p53* pathway can serve as an initiating event in OS, with a subsequent mutation in the *Rb* pathway strongly accelerating tumor development.

These engineered mouse models of OS reproduce many features of human OS including similar gene-transcription signatures (76) and cytogenetic complexity. However, the sites of primary tumor formation in *Cre*–loxP mice do not recapitulate the spontaneous human disease. The majority of lesions (85%) arise in axial skeletal sites (mandibule, maxilla, rib/vertebra, skull, sternum) while on 13.6% of tumors developed from the appendicular skeleton (hind leg, front leg) (68). This contrasts with the anatomic distribution of OS diagnosed in humans, with the distal femur, proximal tibia, and proximal humerus being the most common sites involved and only 10% develop in the axial skeleton, most commonly the pelvis (5). Only in one study (69) did the tumor arise primarily in long bones. In addition, the observed frequency of distant metastases was comparatively low when compared to human disease except for the *P53* knockdown model (69). As opposed to a complete deletion of *P53*, the primary tumor cells proliferated slower and the animals did not have to be sacrificed for local tumor size prior to completion of the metastatic process. Furthermore, the primary site of metastases in human OS is predominantly the lung parenchyma while in *Cre*–loxP mice, sites of metastases were more diverse with both the lung and liver being affected in almost equal proportions.

Other genes such as *C-FOS* (77, 78)*, TWIST* (79)*, p14ARF* (80)*, p16INK4a* (81)*, PRKAR1A* (71), and *p21CIP* (82) have also been implicated in OS pathogenesis based on studies of human OS samples. Their mutation appears to complement the defects in the *P53* and *RB* pathways, and their involvement in osteosarcomagenesis is also demonstrated from genetically engineered mouse models. They provide important information regarding the genetics of OS, but the long latency combined with low penetrance makes utilization of these models less practical.

## Targeted Therapies in OS

Osteosarcoma is very resistant to therapy and therefore there is an urgent need to effectively treat affected patients. The emergence of new anti-cancer drugs and the small number of patients eligible for early-phase clinical trials present another challenge in the clinical testing of novel compounds for OS treatment. As discussed earlier, xenotransplantation models have provided the greatest utility for preclinical screening of drugs with tumoricidal potential. To this end, the National Cancer Institute (NCI) has implemented the Pediatric Preclinical Testing Program (PPTP), a consortium of institutions across the United States and in Australia. Its objective is to identify agents with significant activity in panels of mouse xenograft models representing the most common pediatric cancers including OS (83). The program has been successful, leading to Phase I and II clinical trials for cixutumumab, sorafenib, and rapamycin for OS treatment. (84–86). In each case, these agents demonstrated high levels of response in the PPTP and were well-tolerated with promising anti-tumor activity in some adult and pediatric patients.

The use of spontaneous and transgenic OS models for high throughput screening of anti-OS drugs is hampered due to practical considerations associated with the cost and time of generating sufficient numbers of animals for statistically meaningful data. This is due to variations in disease onset as well as tumor heterogeneity, incidence, and progression. However, the recent generation of transgenic animals expressing *sh*RNAs to knock down *P53* (69) represents a potential breakthrough with respect to preclinical screening. Unlike conventional *Cre*-mediated gene deletion approaches, *P53* knock down mice exhibited 100% penetrance for osteoblastic OS (the most common form of the disease). Moreover, the tumors were most frequently present in long bones and preferentially disseminated to the lungs, consistent with human OS.

Another consideration for preclinical testing in *in vivo* models is the accurate measurement of the disease burden at non-accessible sites. The use of *in vivo* imaging offers the opportunity to detect and monitor the development and progression of the disease. However, imaging systems are costly and not always widely accessible for many researchers. OS has the advantage that the primary tumor in genetically engineered mouse models appears in long bones and is therefore more accessible than abdominal tumors. The monitoring/visualization of micrometastases represents a greater challenge due to their small size. Inaccurate evaluation of metastatic spread in preclinical studies potentially leads to disappointing results in clinical trials. Consequently, there is great interest in refining the methods to enable reproducible and ultrasensitive detection of metastases at the single cell level. The main focus therefore is on techniques, which allow the detection of tumor cells *in vivo*, such as microcomputer tomography (micro-CT), positron emission tomography (PET), bioluminescence, or fluorescence imaging.

## Conclusion

Our understanding of human OS biology is hindered by its rapid onset, low prevalence, and absence of predisposing conditions or precursor lesions. With limited human tissue available for study, animal models provide a valuable tool to investigate the underlying mechanisms driving tumor initiation, progression, metastatic events, and therapeutic interventions. While these models have yet to faithfully recapitulate all aspects of OS, there is no doubt that the study of OS animal models has enabled insight into the genetics of tumor initiation as well as the cellular and molecular profiles of tumor growth and metastasis. In particular, gene knockout studies have been instrumental in identifying genetic mutations that promote OS tumor initiation (*P53)*, as well as co-operative mutations that increase disease incidence (*RB, c-FOS*).

With the use of cell lineage specific markers, it is now possible to introduce genetic mutations by sequential targeting from early precursor (multipotent mesenchymal cell) to more mature osteoblastic cells (osteoblast to osteocyte) to investigate OS incidence and tumor pathology. With this strategy, *Prx1* and *Osx* have been used to identify mesenchymal and osteoprogenitor cells, respectively, following conditional mutation of *P53*. It remains to be seen, however, whether these populations are truly distinct, as *Prx1* could be coexpressed with *Osx* in a certain subpopulation of cells. Another consideration particularly relevant in OS is its tumor heterogeneity among patients, which suggests that multiple cell types could act as cell of origin. Additionally, this concept of heterogeneity calls into question the utility of models exploiting single gene manipulation. Its consideration may permit a more systematic analysis of the genetic lesions involved in OS initiation and progression and could serve as a platform for the identification of early disease biomarkers. Cell of origin identification may also have important implications in the prevention of relapse and elucidate key molecular pathways and driver mutations that could lead to new therapeutic approaches to prevent the disease.

Thus, although for now, conventional orthotopic and subcutaneous transplantation models will remain indispensable to continue the study of OS *in vivo*, new models of spontaneous OS need to be developed to further our understanding of OS biology. Models that accurately reproduce the establishment of spontaneous micrometastases are necessary to investigate novel antimetastatic agents, as this clinical scenario is most often the lethal event for patients with this form of cancer.

## Conflict of Interest Statement

The authors declare that the research was conducted in the absence of any commercial or financial relationships that could be construed as a potential conflict of interest.
