# Corrigendum: Animal models in osteosarcoma

**DOI:** 10.3389/fgene.2014.00475

**Published:** 2015-01-14

**Authors:** Maria V. Guijarro, C. Steve Ghivizzani, Parker C. Gibbs

**Affiliations:** Musculoeskeletal and Oncology Lab, Orthopaedics and Rehabilitation, University of FloridaGainesville, FL, USA

**Keywords:** animal models, osteosarcoma, RB, conditional mouse models, germ-line mouse models, p53

## Conflict of interest statement

The authors declare that the research was conducted in the absence of any commercial or financial relationships that could be construed as a potential conflict of interest.

